# Feasibility and acceptability of an HPV self-testing strategy: lessons from a research context to assess for ability to implement into primary care at a national level in Botswana

**DOI:** 10.3389/fgwh.2023.1300788

**Published:** 2024-01-08

**Authors:** Rebecca Luckett, Doreen Ramogola-Masire, Devon A. Harris, Annika Gompers, Kelebogile Gaborone, Lorato Mochoba, Lapelo Ntshese, Anikie Mathoma, Maduke Kula, Roger Shapiro, Elysia Larson

**Affiliations:** ^1^ Department of Obstetrics and Gynecology, Beth Israel Deaconess Medical Center, Boston, MA, United States; ^2^ Botswana Harvard Health Partnership, Gaborone, Botswana; ^3^ Department of Obstetrics and Gynecology, University of Botswana, Gaborone, Botswana; ^4^ Department of Obstetrics, Gynecology, and Reproductive Biology, Harvard Medical School, Boston, MA, United States; ^5^ Department of Obstetrics and Gynaecology, University of Pretoria, Pretoria, South Africa; ^6^ Department of Obstetrics and Gynaecology, Bamalete Lutheran Hospital, Ramotswa, Botswana; ^7^ National Cervical Cancer Prevention Program, Ministry of Health and Wellness Botswana, Gaborone, Botswana; ^8^ Department of Immunology and Infectious Diseases, Harvard T.H. Chan School of Public Health, Boston, MA, United States

**Keywords:** human papillomavirus (HPV), HPV testing implementation, cervical cancer screening, low-and middle-income countries (LMICs), acceptability, feasibility

## Abstract

**Background:**

The WHO strategy for cervical cancer elimination strives to achieve 70% coverage with high-performance cervical screening. While few low- and middle-income countries have achieved this, high-risk human papillomavirus (hrHPV) self-testing creates the possibility to rapidly upscale access to high-performance cervical screening across resource settings. However, effective hrHPV screening requires linkage to follow-up, which has been variable in prior studies. This study developed and tested an implementation strategy aimed at improving screening and linkage to follow-up care in South East District in Botswana.

**Methods:**

This study performed primary hrHPV self-testing; those with positive results were referred for a triage visit. Withdrawals for any reason, loss-to follow-up between hrHPV test and triage visit, and number of call attempts to give hrHPV results were also documented. Acceptability of the program to patients was measured as the proportion of patients who completed a triage visit when indicated, meeting the *a priori* threshold of 80%. Feasibility was defined as the proportion of participants receiving the results and attending follow-up. To assess the associations between participant characteristics and loss-to-follow-up we used log-binomial regressions to estimate risk ratios and 95% confidence intervals (CI).

**Results:**

Enrollment of 3,000 women occurred from February 2021 to August 2022. In total, 10 participants withdrew and an additional 33 were determined ineligible after consent, leaving a final cohort of 2,957 participants who underwent self-swab hrHPV testing. Half (50%) of participants tested positive for hrHPV and nearly all (98%) of participants received their hrHPV results, primarily via telephone.  Few calls to participants were required to communicate results: 2,397 (82%) required one call, 386 (13%) required 2 calls, and only 151 (5%) required 3–5 calls. The median time from specimen collection to participant receiving results was 44 days (IQR, 27–65). Of all hrHPV positive participants, 1,328 (90%) attended a triage visit.

**Discussion:**

In a large cohort we had low loss-to-follow-up of 10%, indicating that the strategy is acceptable. Telephonic results reporting was associated with high screening completion, required few calls to participants, and supports the feasibility of hrHPV self-testing in primary care followed by interval triage.

## 1 Background

Globally, cervical cancer incidence continues to rise, especially in low- and middle-income countries (LMICs), which account for approximately 85% of new cases and deaths annually (1). The inequity in the burden of cervical cancer is a result of lack of availability of both effective screening and treatment services (2). Cervical cancer is particularly devastating in countries with high burdens of human immunodeficiency virus (HIV), where cervical cancer occurs more frequently and at earlier ages (3). A central pillar of the World Health Organization's (WHO) strategy for cervical cancer elimination is 70% coverage of eligible women with a high-performance cervical screening test twice in their lifetime (4). Many LMICs continue to strive for, but have thus far been unable to achieve this goal (5).

Until recently, the mainstay of cervical screening in high-income countries has been cytology. While this has reduced incidence and mortality from cervical cancer, it is resource intensive, requiring trained staff to perform pelvic examinations, as well as pathology services and administrative capacity to relay results (6–8). Visual inspection with acetic acid (VIA) is an alternative, low-resource method of cervical screening currently employed in many LMICs. It has the benefit of being able to be easily implemented as part of a “See & Treat” model, which allows for recognition and treatment of dysplasia in the same visit, however, it is subjective and accuracy of results are highly variable (9). In addition, VIA as a primary screening method requires specialized training of every provider, which is often time consuming and expensive, and still requires pelvic examinations for all women. Unfortunately, issues with infrastructure and resources often inherent to LMICs have made it difficult to adequately upscale either VIA or pap smear to reach target population coverage (10).

In recent years, primary screening with high-risk human papillomavirus (hrHPV) testing has become the global standard for high-performance testing. It has the highest sensitivity for detecting cervical dysplasia and is the most effective primary screening strategy available (11, 12). Several studies have validated the use of primary hrHPV testing for population level screening in high-risk groups, including women living with HIV (WLHIV), while also demonstrating that self-collection of hrHPV swabs is non-inferior to provider collection and is the most cost-effective approach in the long-run to addressing the issues stated above (13–15). High-risk HPV testing is thus a highly attractive option for screening in LMICs as little clinical infrastructure is needed to collect HPV self-swabs. Additionally, vaginal self-swabs spare patients a pelvic examination while allowing hrHPV testing at various patient-provider interactions, reducing the need for skilled professionals and improving access. Though it does require a laboratory set-up, most LMICs have laboratory capacity that may be leveraged to support the introduction of HPV-based cervical screening programming, thus mitigating the issues with upfront affordability that have some questioning the cost-effectiveness of this method (16).

A major challenge of primary hrHPV screening is the low positive predictive value for cervical dysplasia, and positive results require linkage to follow-up triage and/or treatment according to national guidelines (17, 18). In studies in other settings, adherence to triage follow-up has been variable and often low, which impacts the effectiveness of HPV-based screening programs, and the ideal triage strategy from a cost-effectiveness standpoint has yet to be determined and likely varies by health system (15, 19–21).

This study developed and tested an implementation strategy in a research context aimed at introducing primary hrHPV screening and linkage to follow-up care in Botswana. We evaluated implementation outcomes of the strategy, with a focus on acceptability and feasibility. This research aimed to be at the forefront of the WHO elimination strategy's secondary screening pillar bolstering equitable access to high-performance cervical screening for all women, including those women at highest risk of cervical cancer – WLHIV in LMIC's.

## 2 Materials and methods

### 2.1 Implementation context

Cervical cancer is the most common cancer in Botswana, and the leading cause of cancer-related death. In 2020 in Botswana, 31.9% of all cancer diagnoses amongst women were cervical, and women have a 3.5% lifetime risk of cervical cancer compared to 0.7% in the United States (22, 23). The relationship between cervical cancer and the high population prevalence of HIV remains clear - although only 22% of reproductive-aged women are living with HIV in Botswana, 69% of cervical cancer cases occur in WLHIV (24–26). While Botswana has led the region in progressive HIV treatment policy, with nearly 90% antiretroviral therapy (ART) coverage of the known HIV-infected population, cervical screening has not kept pace (27).

Cervical screening with cytology was introduced in government-run health facilities in 2000, and in 2014 services were expanded to include “See & Treat” (SAT) programming with VIA and linkage to cryotherapy and loop electrosurgical excision procedure (LEEP). Screening services were organized in a hub and spoke model with spokes being multiple peripheral sites offering VIA and cytology screening and referring clients requiring treatment or more complex diagnosis to LEEP hubs. At the beginning of 2021 there were 57 VIA sites and 35 LEEP sites in Botswana. Despite the wide distribution of services and the fact that cervical screening diagnostic and treatment services are available free to citizens, only 31% of the target population accessed cervical cancer screening between 2017 and 2021 (Ministry of Health and Wellness of Botswana (MoHWB, programmatic data) (28).

In 2020, the NCCPP drafted the new national cervical cancer prevention strategy for 2021–2025, which introduced HPV-based screening algorithms into national cervical screening programming, with a goal to move toward universal hrHPV testing over five years. Following pilot demonstration projects, the National Cervical Cancer Prevention Program (NCCPP) introduced primary hrHPV screening at two sites using Xpert HPV, yet few women had the opportunity to do hrHPV screening due to machine maintenance issues, lack of cartridges, and service burden on the platform from other disease diagnostic services (i.e., Tuberculosis).

Botswana's effective HIV treatment program includes routine HIV viral load testing effected through a regional referral system from health facilities to laboratories with high through-put PCR testing capacity. This referral system and equipment could be leveraged to introduce a highly efficient hrHPV testing program.

### 2.2 Implementation strategy and study participants

This study implemented primary hrHPV self-testing in a research context across one district in Botswana, in collaboration with the MOHWB. Prior to initiation of this study, we met with the local Chief of South East District, Kgosi Mosadi, who facilitated dissemination of information about cervical cancer as a public health issue and screening with hrHPV testing to the sub-chiefs and headman, and ultimately to the community.

A study team was assembled and included three physicians, a part-time study coordinator, 4 full-time research assistants, and a data entry clerk. All research assistants held qualifications in general counseling and HIV testing and counselling. The research team developed language-concordant study materials in both English and Setswana for counseling and education around cervical cancer, cervical cancer prevention, and hrHPV testing. Posters identifying the study and its goals were placed throughout South East District in health facilities, schools, shopping complexes, and the Kgotla (traditional village meeting place).

Between February 2021 and August 2022, 3,000 study participants were recruited from 8 health centers within South East District, Botswana. To better evaluate triage strategies among WLHIV, the cohort was enriched with a goal to recruit 50% WLHIV. Purposeful recruitment was on the highest volume clinical days (which normally corresponded to the day a doctor was providing consultations at the clinic) and on HIV clinic days. The study team provided health education talks in waiting areas of these health facilities and to women working nearby the health centers, and directed interested women to private locations in the respective health facility for further information, confirmation of eligibility, informed consent and enrollment.

Inclusion criteria were non-pregnant women aged 25 years or older with an intact cervix who were able to provide consent. Patients were ineligible if they had previously had a total hysterectomy or had a history of cervical cancer. Participants underwent informed consent in their preferred language of either Setswana or English. After consent, demographic and contact information was obtained by the research assistant, including telephone number, WhatsApp number and a next of kin telephone number.

Once participants were enrolled by research staff, they were then instructed in detail about how to self-collect the vaginal hrHPV swab and provided with a kit containing both a sampling device and storage vial. They were directed to the facility bathroom for self-collection and specimens were then returned to the research assistant who ensured appropriate labeling of the specimen and completion of the lab requisition form. Specimens were then kept refrigerated and transported to the research lab by research assistants once weekly. The specimens were tested using the AmpFire® HPV Assay (Atila BioSystems, Mountain View, California, USA) for 15 hHPV types (including HPV 16, 18, 31, 33, 35, 39, 45, 51, 52, 53, 56, 58, 59, 66, 68) (29).

**Figure 1:**
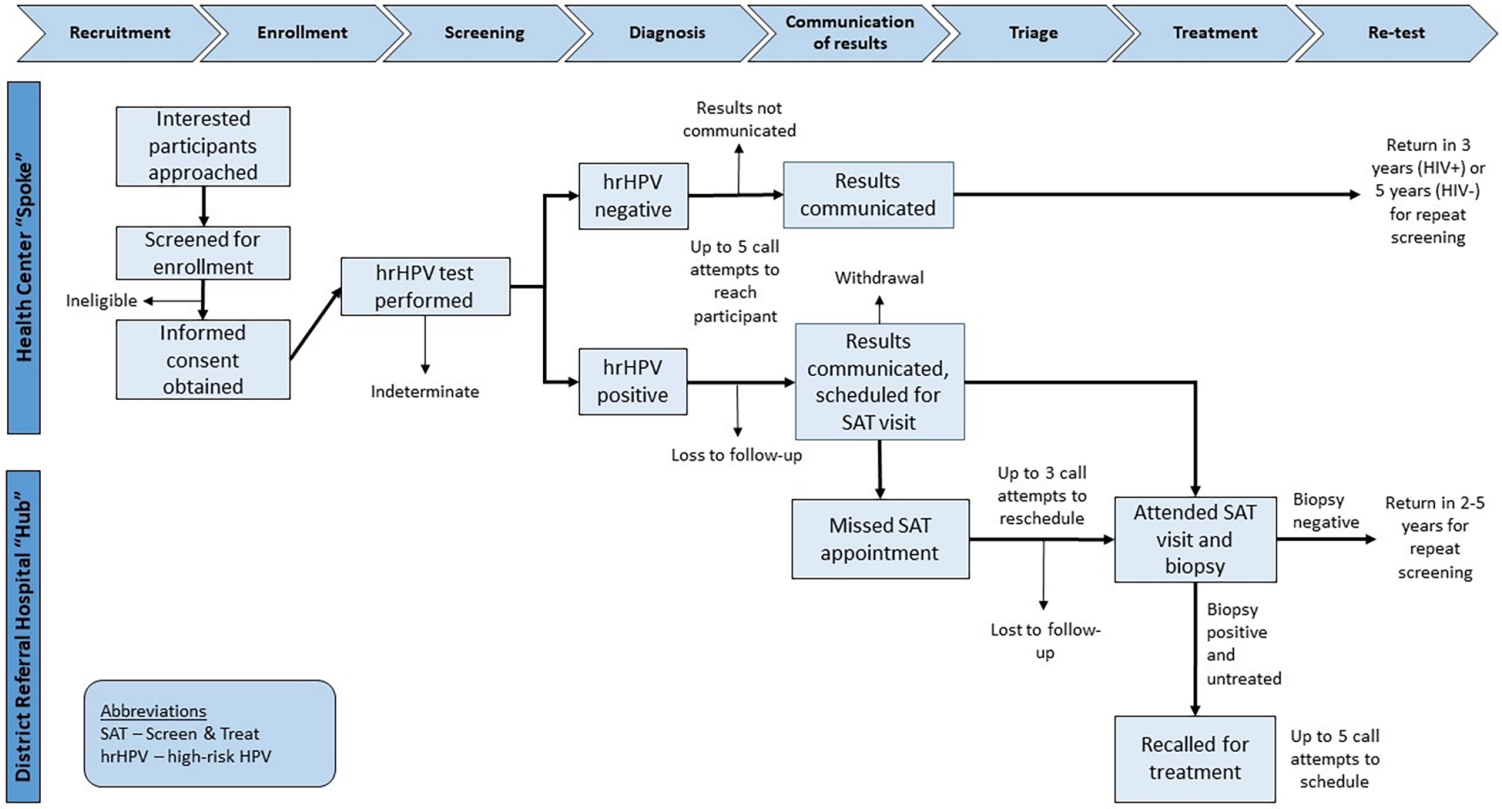
Cascade of care throughout study procedure.

The electronic laboratory information system was checked by study staff once weekly for hrHPV test results, and when obtained those results were entered into the Research Electronic Data Capture (REDCap) system, which is a HIPAA-compliant, web-based data collection tool (30). Participants (or next of kin) were then called regarding their results. At the time of contact, those participants with positive hrHPV results were scheduled for a triage visit at the existing hub site corresponding to the spoke clinic from which they were recruited. No compensation was offered to participants for attending triage visits. Five call attempts, on separate occasions, were made to the study participant to attempt to relay results and schedule follow-up, if needed. For those who missed their scheduled appointments, an additional 3 call attempts on separate occasions were made to study participants in an effort to reschedule them for their triage visit. The flow of study procedures from enrollment to follow-up is presented in Figure 1.

### 2.3 Outcomes and measurement

The Proctor Implementation Outcomes framework guided the choice of constructs used to evaluate the implementation strategy (31). A description of all implementation and service measures is included in Table 1.

**Table 1:** Evaluation of the hrHPV implementation strategy using the taxonomy of the proctor implementation outcomes framework (31).

	Outcome	Outcome measure	Result
Implementation outcomes	Acceptability	Percent adherence with triage (hrHPV+)	1,327/1,478 (90%)
		Percent loss to follow-up at any point (hrHPV+)	150/1,478 (10%)
		Median time between test result and communication to participant	17 days (IQR, 5–35)
		Median time between participant receipt of results and triage visit, when indicated	7 days (IQR, 4–20)
		Participant willingness to receive results via telephone	2,946/2,957 (99.6%)
	Feasibility	Number women screened per research assistant (over 1 year)	739 participants
		Median time between specimen collection and health care worker receipt of results	20 days (IQR, 9–37)
		Proportion of participants receiving hrHPV results	2,907/2,957 (98.3%)
		Median number of call attempts to provide hrHPV results to participants	1 (IQR, 1–1)
	Penetration	Increase in the number of screening facilities	3 facilities to 8 (267%)
		Increase in the number of women screened per 6 month period	283–1,458 (515%)^a^
Service Outcomes	Effectiveness	Proportion of women with CIN2 or CIN3 who were appropriately treated	193/197 (98%)
		Proportion of women with invasive cancer or adenocarcinoma in-situ who were appropriately referred	9/9 (100%)
	Timeliness	Median time between participant enrollment/test and participant receiving test results	44 days (IQR, 27–65)

All data presented as proportion (percent) or median ([interquartile range (IQR)].

^a^ Excludes participants from sites E and F due to missing data regarding pre-implementation screening numbers.

Most individual implementation and service outcomes were documented in REDCAP by research assistants. One measure of acceptability, participant willingness to receive hrHPV test results by phone, was measured using an interviewer-administered survey, documented in REDCap, at the time of enrollment. Penetration was measured by the increase in the number of facilities offering cervical screening within South East District, defined as any government health center in the district offering VIA or pap smear, well as increase in the number of women screened during study implementation, defined as any woman receiving those services over the same time frame. Baseline values for both were measured through South East District Health Management Team (DHMT) Internal Reports from May through October 2019 (2020 was excluded due to COVID impacts on health systems and screening) and compared to the same time frame in 2021 during study implementation.

In addition to the above implementation outcomes, the service outcomes of effectiveness and timeliness were also evaluated. Effectiveness was measured as how many women with cervical intraepithelial neoplasia (CIN) 2 or worse [considered CIN2+, which includes CIN2, CIN3, CIN3 with microinvasion, adenocarcinoma in-situ (AIS), and invasive carcinoma] received treatment or were referred for further management. Timeliness was measured as the duration of time between participant enrollment and communication of HPV test result, as well as receipt of HPV results rand triage visit (when applicable).

To determine if the intervention was acceptable, several indicators were assessed including the proportion of patients who received their hrHPV results, and who completed a triage visit when indicated by a positive hrHPV screening test. We considered the *a priori* threshold of 80% as indicating acceptability. All additional implementation and service outcomes are reported without *a priori* thresholds to qualify them.

### 2.4 Analysis

Descriptive statistics are presented as the frequency (percentage), mean (± standard deviation), or median (interquartile range), as relevant. To assess the associations between participant characteristics and loss-to-follow-up we used log-binomial regressions to estimate risk ratios and 95% confidence intervals (CI). Data were analyzed with SAS 9.4 (SAS Institute Inc., Cary, NC, USA).

### 2.5 Ethics

The institutional review boards of the Botswana Ministry of Health and Wellness (13/18/1), the University of Botswana (URB/IRB/1543), Beth Israel Deaconess Medical Center (2019P001130) and the South East District Health Management Team approved this study. The studies were conducted in accordance with the local legislation and institutional requirements. The participants provided their written informed consent to participate in this study. The study is registered on Clinicaltrials.gov (NCT04242823).

## 3 Results

### 3.1 Patient characteristics

Enrollment of 3,000 women in this study occurred from February 2021 to August 2022. In total, 10 (0.3%) participants withdrew and an additional 33 (1.1%) were determined ineligible after consent, leaving a cohort of 2,957 women who underwent self-swab primary hrHPV screening.

Demographics for the study population are reported in Table 2. Participants had a mean age of 42; the majority were parous (92%), and identified as single (72%). Half (*n* = 1,479) of participants were HIV positive. Half (*n* = 1,478) of participants tested positive for at least one hrHPV type. Three women had indeterminate results on initial hrHPV test and returned for repeat hrHPV self-swab.

**Table 2:** Demographic and health characteristics of 2,957 women who underwent HPV testing in South East district, Botswana.

Characteristic	*N* (%) *n* = 2,957
Age, years ± standard deviation	42 ± 11
Education	
≤Primary	562 (19)
≥Secondary	2,395 (81)
Employed	1,672 (57)
Marital status	
Single	2,118 (72)
Married	674 (23)
Divorced/Separated	38 (1)
Widowed	127 (4)
Gravidity	
0	190 (6)
1–3	1,897 (64)
≥4	870 (29)
Parity	
0	222 (8)
1–3	2,056 (70)
≥4	679 (23)
Age of sexual debut, years ± standard deviation	19 ± 3
Lifetime sexual partners^a^	
0	4 (0.1)
1–5	1,940 (66)
≥6	995 (34)
Smoking	172 (6)
Health facility where HPV test performed	
Hospital	1,122 (38)
Clinic	1,835 (62)
History of cervical cancer screening	1,989 (67)
HIV positive	1,479 (50)
High-risk HPV positive	1,478 (50)

Data presented as *n* (%) or mean ± standard deviation.

^a^ Excluding 18 participants with missing data; data collected via interviewer-administered survey with categorical response choices.

### 3.2 Results communication

Nearly all [2,907 (98%)] participants tested received their hrHPV results, including 98% of hrHPV positive women (*n* = 1,448). The majority of participants received their results by phone (99%), with few receiving them in person (3%), or via SMS/WhatsApp (<1%).

The median number of days from specimen collection to laboratory publication of hrHPV result was 20 days (IQR, 9–37). The median number of days from publication of results to participant receiving results was 17 days (IQR, 5–25). Overall, few calls to participants were required to communicate hrHPV test results: 2,397 (83%) required one call, 386 (13%) required 2 calls, and only 151 (5%) required 3–5 calls. At the time of communication of results, hrHPV positive participants were counseled on the need for triage of positive results and attempts were made to book patients into hub LEEP clinics for visual assessment and treatment according to “See & Treat” (SAT) protocols. The median time between communication of positive hrHPV result to participant and participant presentation to triage visit was 7 days (IQR, 4–20).

### 3.3 Loss to follow-up

**Figure 2:**
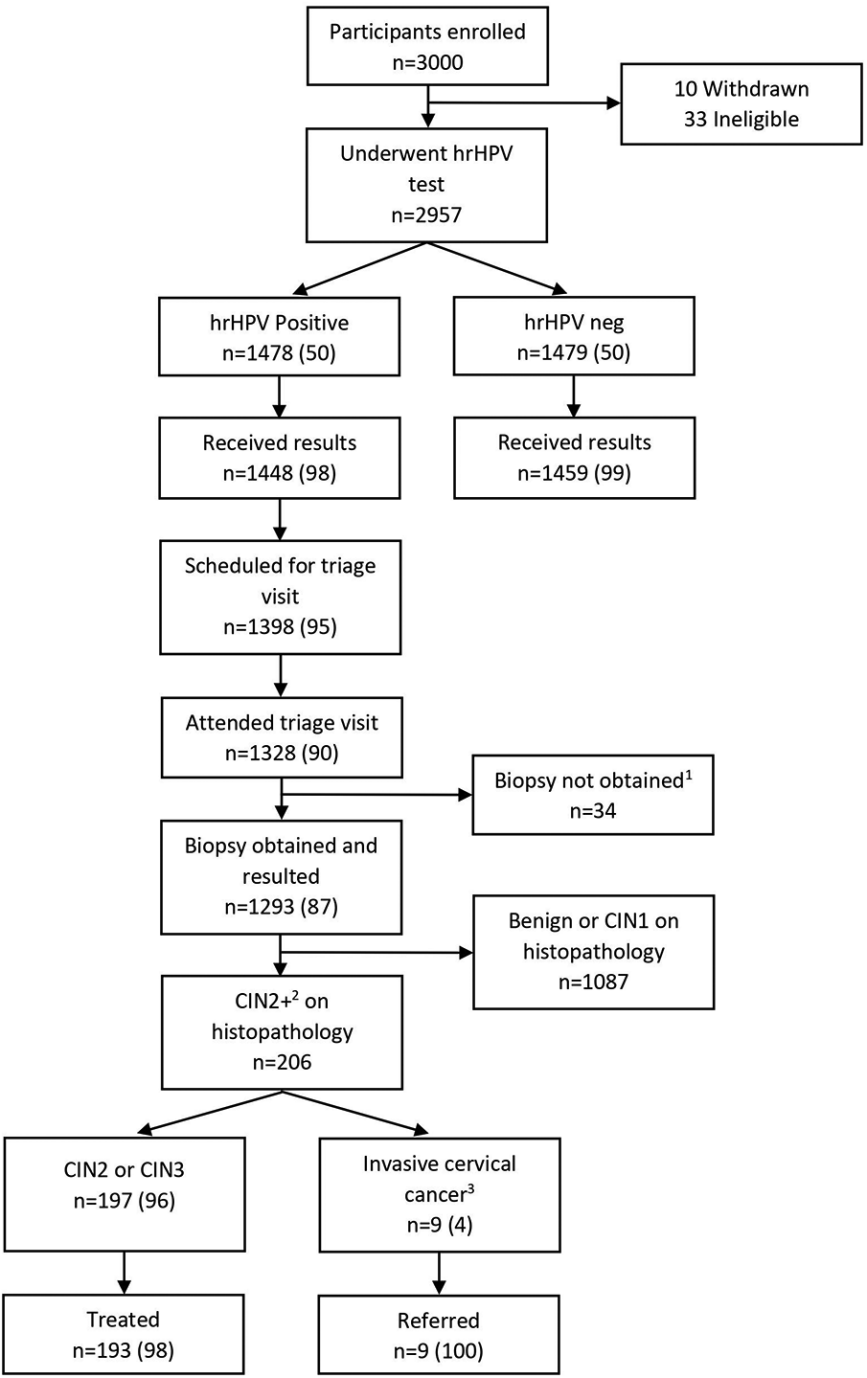
Participant flow diagram through study. All data presented as *n* (%). ^1^10 specimens inadequate for interpretation in the laboratory, 7 specimens lost in the laboratory, 15 results not yet reported by the laboratory, 2 specimens not collected due to anatomic issues. ^2^Includes CIN2, CIN3, CIN3 with microinvasion/invasive features, adenocarcinoma *in situ*, and carcinoma. ^3^Includes CIN3 with microinvasion/invasive features, adenocarcinoma *in situ*, and carcinoma.

Of the 1,478 hrHPV positive participants, 1,328 attended a triage SAT visit, resulting in a loss to follow-up of 10% (*n *= 150). Of those who did not return, 30 patients were unable to be contacted regarding their test results, 50 participants declined return for triage appointment, and 70 participants were scheduled for but did not keep their triage appointments. Of those who attended a triage visit, 97% (*n *= 1,293) had a biopsy obtained for histopathologic diagnosis. For those who did not have a biopsy result available for analysis, 10 had inadequate specimen, 2 had no specimen collected, 7 had a lost specimen, and 15 had histopathologic results still pending at the time of analysis. This cascade of care, with loss to follow-up at each point, is shown in Figure 2.

Participant characteristics associated with loss to follow-up are shown in Table 3. Increasing distance to nearest triage site was associated with higher loss to follow-up; each 1 km increase in distance was associated with a 1.2 times increased risk of loss to follow-up (95% CI, 0.97–1.4). Additionally, WLHIV and those with no history of prior cervical screening were more likely to be lost to follow-up [RR, 1.5 (95% CI, 1.1–2.0) and 1.6 (95% CI, 1.2–2.2), respectively]. Meanwhile, marriage was a weakly protective factor [RR, 0.57 (95% CI, 0.36–0.90)].

**Table 3:** Factors associated with loss to follow-up amongst 1,478 hHPV positive participants in South East district, Botswana.

Characteristic	Total hrHPV + participants (*n* = 1,478)	Loss to follow-up (*n* = 150)	RR (95% CI)
Distance to nearest triage site (per km)			1.18 (0.99–1.42)^a^
Recruitment Site (distance to triage site)			
Site A (0 km)	533	21 (4)	Ref
Site B (4 km)	129	10 (8)	1.97 (0.95–4.1)
Site C (8 km)	169	12 (7)	1.80 (0.91–3.6)
Site D (8 km)	97	11 (11)	2.88 (1.4–5.8)
Site E (13 km)	146	28 (19)	4.87 (2.9–8.3)
Site F (15 km)	82	14 (17)	4.33 (2.3–8.2)
Site G (36 km)	224	44 (20)	4.99 (3.0–8.2)
Site H (52 km)	98	10 (10)	2.59 (1.3–5.3)
Education level^b^			
Secondary/Tertiary	1,201	124 (10)	Ref
None/Primary	277	26 (9)	0.91 (0.61–1.4)
Employed			
Yes	844	78 (9)	Ref
No	634	72 (11)	1.23 (0.91–1.7)
Marital Status			
Single	1,088	123 (11)	Ref
Married	310	20 (7)	0.57 (0.36–0.90)
Divorced/Separated	17	3 (18)	1.56 (0.55–4.4)
Widowed	63	4 (6)	0.56 (0.21–1.5)
HIV status^c^			
Negative	649	52 (8)	Ref
Positive	823	97 (12)	1.47 (1.1–2.0)
Have you been screened in the past?^d^			
Yes	957	79 (8)	Ref
No	520	70 (14)	1.63 (1.2–2.2)

^a^ Risk of loss to follow-up per 1 km increase in distance to nearest triage site.

^b^ Excludes 1 participant who attended special education.

^c^ Excludes 6 participants unsure of HIV status.

^d^ Excludes 1 participant unsure of prior cervical cancer screening status.

### 3.4 Increased capacity

Reliable pre-implementation screening data exists for 6 of 8 clinics in the South East District. When comparing the number of women screened at these 6 sites from May through October 2019 (*n* = 283) to May through October 2021 (*n* = 1,458) this represents a 515% increase in number of women screened over this 6-month time frame after implementation (MOHWB Internal data, 2019). Given the impacts of the COVID-19 pandemic on the healthcare system in Botswana, 2020 data was not available. Additionally, with a total of 4 research assistants (with training equivalent to healthcare auxiliaries) there were 739 women screened per non-MD staff member over the entire 18-month study period.

Prior to project implementation there were 3 designated cervical screening site in South East District; by moving to primary hrHPV testing this increased to 8 screening sites and maintained the already existing referral pattern to designated hub sites for triage.

## 4 Discussion

This study showed that the introduction of high-performance screening is not only technically within reach of LMICs but also that implementation is feasible and acceptable within existing health infrastructure in Botswana. While this work was performed as research, it was done collaboratively with the MOHWB within existing government healthcare systems with success. We performed high volume screening with the support of existing cervical screening teams, increasing penetration of cervical screening services across all health facilities in the district (from 3 to 8 facilities) and bringing primary screening closer to women. This was accomplished with the addition of only 4 research assistants working full-time across the eight facilities, over 10 months, and then part time for the following 8 months. These research assistants had the technical expertise equivalent to healthcare auxiliaries and were able to facilitate counseling and collection of specimens. Thus, our findings demonstrate we should not only train and engage nurses and doctors in cervical cancer screening but also create specific training for health care auxiliaries and other community health workers who, in the future, could absorb this role in collaboration with existing cervical cancer screening nurses. The time saved with primary HPV testing as compared to performing primary screening with either VIA or Pap smear, which both require pelvic examinations, can be utilized to increase HPV-based screening while also supporting triage visits for those with positive hrHPV results.

Our strategy of telephonic communication of hrHPV results and interval triage of positive results was implemented successfully. Nearly all women received their HPV results (98%) and a majority of women required only one telephonic communication to receive their results. Telephonic communication of results was deemed acceptable by a majority of women, and if implemented would save healthcare workers the increased time associated with in-person clinical encounters currently used for communication of results. Furthermore, once women received their results, the median time to move through the cascade of care to triage was only one week, indicating that interval triage was acceptable to women.

Loss to follow-up between primary cervical screening and triage was relatively low compared to other settings and well within accepted standards across settings (21). Our loss to follow-up of 10% is particularly striking compared to loss to follow-up in a study in Kenya where hrHPV testing aimed to be point-of-care and achieved an average test turn-around time of 24 h, yet had loss to follow-up of 90% (32). Our findings support implementation of a test and recall strategy in our setting, which can replace less clinically effective “See & Treat” (SAT) services until the time that a true point-of-care hrHPV test is available to allow for same day test-and-treat services. It also supports the possibility of integrating HPV-based cervical screening into primary care services or at multiple points of contact within the healthcare system, with the support of task-sharing and clear pathways to interval triage.

When planning programming, it will be useful to make efforts to mediate factors associated with increased loss to follow-up. Distance between screening and facilities was the most notable and could potentially be ameliorated by providing transportation, or increasing triage sites in more remote communities. Surprisingly, WLHIV had slightly increased loss to follow-up, despite these participants likely having increased contact with the healthcare system, particularly in Botswana which employs a universal treatment policy. Women who had never been previously screened were also more likely to be lost to follow-up. This speaks to the need for increased counseling around the role and importance of screening, the relationship between HIV and cervical cancer, as well as general support, education, and encouragement from the health system to return. Marital status was weakly protective against loss to follow-up, which may be related to underlying factors such as age, socioeconomic security, and social supports.

Although our laboratory turn-around time was relatively long, impacting the overall time between hrHPV test and triage (44 days), it is likely that systematization of hrHPV testing would result in improved turn-around times as has occurred with other programs, such as HIV viral load testing. We recommend that systems develop clear laboratory pathways and establish expected turn-around times when implementing primary hrHPV screening. In fact, Botswana can leverage its successful HIV viral load testing program, with distributed PCR platforms across the country and existing referral pathways and specimen transport systems to support successful implementation of primary hrHPV screening. In this research we utilized AmpFire HPV which is low cost and PCR platform agnostic. AmpFire had an excellent clinical performance with a low invalid rate (0.13%). Ultimately, however, this time interval would not significantly impact clinical outcomes for women (33).

There were other learnings from implementation of this program that were less measurable but noted by the implementing team. The first was how primary hrHPV screening increased the volume of women screened in the district and that provided a new motivational energy to the cervical screening staff. Additionally, both nursing staff and research assistants found that word of mouth dissemination of primary hrHPV screening augmented health education in clinics. Women who had participated in screening often returned with friends and family members, even from very distant districts. We also discovered opportunities for improvement in existing systems that would contribute to successful implementation of primary hrHPV screening. Our system for obtaining, recording and communicating hrHPV results was manual. In an optimal program, results notification could be automated to ensure that healthcare staff are not the only bridge connecting patients with their results and triage visits. Data are needed on acceptability of automated, electronic technologic solutions that would facilitate this process and contribute to establishment of a national registry.

Our study was not without limitations. First, this was done in a research setting with additional staffing support in the district. While we do believe that the lessons from the study setting are directly translatable and implementable with the existing staffing complement in the government facilities, directed training and support will be required to successfully launch this into the national program. Second, our data are specific to our middle-income setting with excellent roads, transportation systems, cell phone and network coverage. These factors may impact success in other settings or even in more rural parts of Botswana. Thirdly, we do not have client outcomes data; in the future it will be important to look at participants' satisfaction with the implementation framework.

As the world moves toward primary hrHPV screening as the most sensitive high-performance method of cervical screening, further research in unique contexts is necessary to explore factors that will support successful implementation and achieve population-level screening across settings. Our data suggests that with minimal increased staffing requirements, telephonic linkage to triage, and leveraging of pre-existing laboratory systems primary hrHPV screening is a feasible and acceptable method of cervical cancer screening. Research must be conducted in true collaboration with relevant Ministries of Health and healthcare implementing partners to achieve success. The practical framework used here may support these efforts in other contexts.

## Data Availability

The raw data supporting the conclusions of this article will be made available by the authors, without undue reservation.
